# Unilateral Scissor Bite Managed with Prefabricated Functional Appliances in Primary Dentition—A New Interceptive Orthodontic Protocol

**DOI:** 10.3390/children8110957

**Published:** 2021-10-24

**Authors:** Chaypat Simsuchin, Yong Chen, Shuying Huang, Sreekanth Kumar Mallineni, Zhihe Zhao, Urban Hagg, Colman McGrath

**Affiliations:** 1 Postgraduate Certificate in Orthodontics, Director of Ortho-Kids Club, Chiang Mai 50200, Thailand; toothandgumcm@gmail.com; 2 Department of Stomatology, School of Medicine, Xiamen University, Xiamen 361005, China; shuyinghuang@yeah.net; 3 Department of Preventive Dental Science, College of Dentistry, Majmaah University, Al-Majmaah 11952, Saudi Arabia; drmallineni@gmail.com; 4 Department of Pediatric and Preventive Dentistry, Saveetha Dental College and Hospital, Saveetha Institute of Medical and Technical Sciences, Saveetha University, Chennai 600077, India; 5 Department of Orthodontics, West China School of Stomatology, Sichuan University, Sichuan 610017, China; zhaozhihe@vip.163.com; 6 Discipline of Orthodontics, Faculty of Dentistry, The University of Hong Kong, Hong Kong 999077, China; euohagg@hku.hk; 7 Discipline of Dental Public Health, Faculty of Dentistry, The University of Hong Kong, Hong Kong 999077, China; mcgrathc@hku.hk

**Keywords:** functional appliance, crossbite, scissor bite, interceptive orthodontics

## Abstract

A unilateral scissor bite is a type of severe malocclusion in primary dentition that can influence the development of the mandible and face. The present case report describes an interceptive treatment on a 4-year-old girl with a unilateral scissor bite combined with an anterior crossbite, increased reverse overjet, and overbite on a skeletal Class III base. The patient was treated with a new Prefabricated Functional Appliance (PFA) which was modified from a Frankel-III appliance to induce a mandible to move backwards and relieve an occlusion discrepancy of the dental arch, and a functional dental rehabilitation with resin and myofunctional exercises with a PFA which was modified from an oral screen appliance were performed during the treatment. The total treatment time was 11 months and a satisfactory improvement of occlusion and facial esthetics was achieved. This case report presents a new PFA system and treatment protocol in primary dentition which results in a favorable outcome, and the clinical effectiveness of this appliance system warrants further investigation.

## 1. Introduction

A unilateral scissor bite is a severe malocclusion which is usually accompanied by a lateral functional shift and displacement of the mandible from the initial contact to maximum intercuspation in primary dentition. It can affect the development of the mandible and face, causing asymmetric growth that results in masticatory and aesthetic problems with considerable impact on quality of life [1,2]. The early intervention of a scissor bite in the primary dentition stage may prevent skeletal asymmetry and subsequent malocclusions in permanent dentition [3]. There are several ways to manage a scissor bite such as the early transverse development of the mandibular arch with fixed or removable appliances in mixed dentition or a surgical approach [4,5].

A Prefabricated Functional Appliance (PFA) is a type of silicone prefabricated removable appliance which combines the characteristics of functional appliances and a positioner [6,7,8,9]. A widely used type of PFA is the Eruption Guidance Appliance (EGA), which was developed by Bergersen to reduce the overbite and overjet, and relieve crowding in early mixed dentition [10,11,12]. A more recent development is the PFA modified from a Frankel appliance, and it has worked well in phase one management prior to phase two with fixed appliance therapy [13]. PFA treatment appears to have some clinical advantages such as comfort in wearing and correcting sagittal, transversal, and vertical relationships simultaneously [7,10].

The aim of the present case report is to describe a new treatment protocol for patients in primary dentition with Class III incisor relationships and a unilateral scissor bite using a novel PFA combined with dental rehabilitation and myofunctional exercises.

## 2. Case Report

### 2.1. History

A 4-year-old female patient visited the orthodontic clinic with a chief complaint of the inability to chew food on the right side of her mouth. She had a clear medical history and without a medication allergy.

### 2.2. Assessments

An extraoral examination showed a concave profile with slightly reduced lower facial proportions. On the assessment of the centric occlusion (CO) and centric relation (CR) discrepancy and guidance of the mandible on closure, a functional shift of the mandible was associated with the lower facial asymmetry. An intraoral examination revealed a complete unilateral scissor bite on the right side combined with an anterior crossbite, and an increased overbite on the skeletal Class III base. The left second primary molars were in a mesial step terminal plane relationship. Tongue thrusting was presented with anterior teeth spacing, and articulation disorders of/s/,/n/,/l/, and/t/were found in the patient [14]. The patient’s Dental Health Component (DHC) of the Index of Orthodontic Treatment Need (IOTN) rating was grade 4 h, which means a severe irregularity, requiring treatment [15]. A behavior assessment was conducted using Frankl’s behavior rating scale, and the patient was rated as definitely positive (good rapport with the dentist, interested in the dental procedures, laughing, and enjoying) [16].

In the panoramic radiograph, she was in the primary dentition stage, all permanent teeth were present except for the third molars. The pre-treatment cephalometric analysis presented with mild Class III skeletal relationships (ANB 0 degree), a high negative value of Wits measurement (−4.5 mm), and the lower incisor inclination was 20 degrees to the NB. Linear measurements showed the maxilla was retrusive with the Nasion perpendicular to point A (−2.7 mm). She had the feature of a “strong chin” appearance—the lower lip was protruded relative to the upper lip.

### 2.3. Treatment Goals

The treatment goals were to correct the anterior crossbite and right scissor bite (to eliminate the CR–CO discrepancy), to normalize the occlusal relationship and improve facial esthetics. In addition, the treatment objectives also included to break the tongue thrusting habits and reduce the asymmetric muscular strain, allowing for a more favorable skeletal facial growth and development.

### 2.4. Treatment Planning

**Figure 1:**
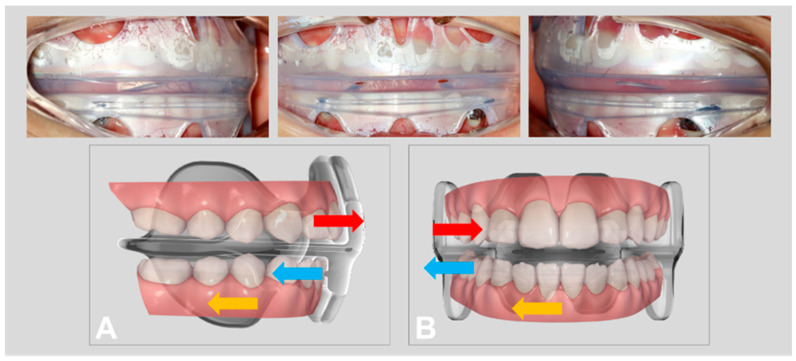
The treatment mechanisms of NOA™-F3 for anterior crossbite. (**A**): Procline upper incisors (red arrow), retrocline lower incisors (blue arrow), and guide mandible backward (brown arrow) and scissor bite. (**B**): Lingually moving maxillary posterior teeth (red arrow), buccally uprighting mandibular posterior teeth (blue arrow), and guide mandible to the left side (brown arrow).

**Figure 2:**
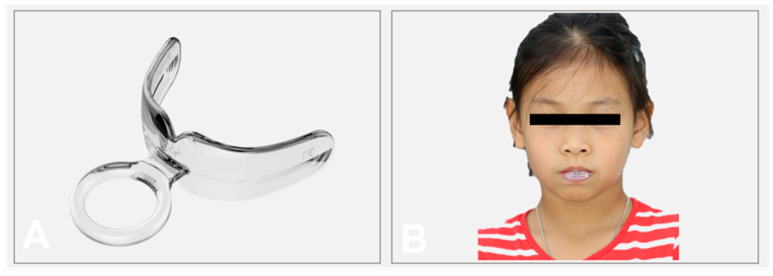
NOA™-T: (**A**) the vertical view; (**B**) the extraoral view.

The treatment plan was to induce the mandible to move backward and relieve the occlusion discrepancy of the dental arch with a functional appliance (NOA™-F3) first, combined with myofunctional exercises to break the tongue thrusting habits, and reduce the asymmetric muscular activities with a prefabricated oral screen (NOA™-T) at the same time. The treatment mechanisms of NOA™-F3 for an anterior crossbite and scissor bite are presented in Figure 1. The vertical view and extraoral view of NOA™-T are presented in Figure 2. Once the maxillary and mandibular primary incisors displayed an edge-to-edge bite relationship, a functional dental rehabilitation with a composite inclined plane on an upper anterior segment and the indirect filled technique with the composite resin on posterior teeth were performed to establish a normal occlusion. The patient and her parents provided consent for the publication of data and images related to her treatment for scientific purposes.

### 2.5. Treatment Sequence

The PFA (NOA™-F3, size M1) was used to open the reverse bite, induce the mandible to move backwards, and coordinate masticatory muscles and temporomandibular joints. The patient was instructed to use the NOA™-F3 every night and 2 h during the daytime, 12–14 h in total to correct the CR–CO discrepancy. Moreover, she was instructed to perform myofunctional exercises (to pull the oral screen forward and backward with a finger) with NOA™-T (size M), which consists of a ring pull and a vestibular shield 10 min a day. The patient and her parent were asked to take photos or videos during these exercises to confirm her treatment compliance.

After 2 weeks of treatment, the maxillary and mandibular primary incisors displayed an edge-to-edge bite relationship, the resin-filled celluloid crowns on 51 and 61 were created with thickened composite inclined planes. At the 3-month follow-up, the anterior crossbite was corrected, and the composite resin was added on the posterior teeth and resin-filled celluloid crowns on 52 and 62 were created.

## 3. Results

During the treatment, the patient’s muscle tenderness, discomfort of TMJ, mastication, range of the mandibular movements, swallowing, and speech were evaluated. An improvement in mastication, speech, and facial esthetics confirmed the patient’s tolerance of the new mandibular position.

**Figure 3:**
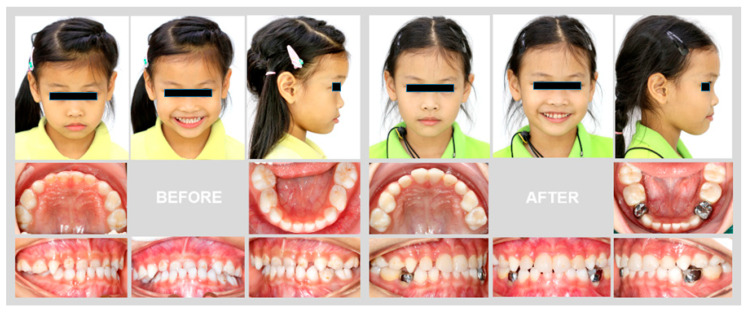
Pre-treatment and post-treatment extraoral and intraoral photographs.

**Figure 4:**
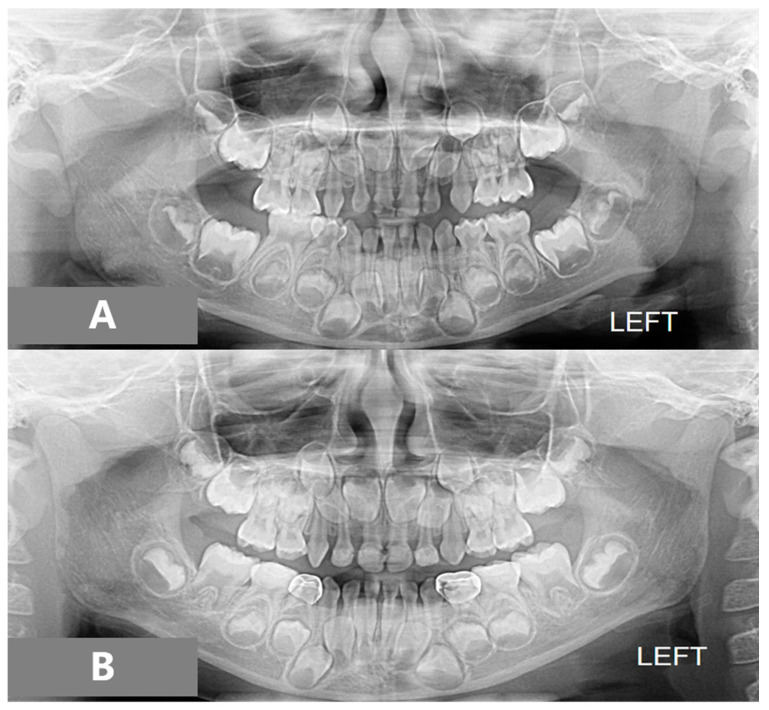
Panoramic radiographs: (**A**) pre-treatment, (**B**) post-treatment.

**Figure 5:**
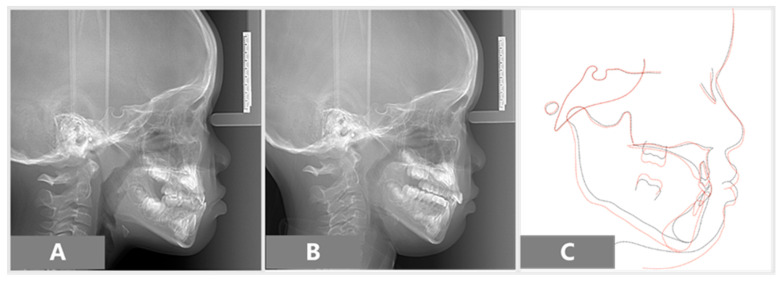
Lateral cephalograms: (**A**) pre-treatment, (**B**) post-treatment, (**C**) superimpositions of pre-treatment (black line) and post-treatment (red line).

After 11 months of active treatment, the crossbite of the anterior teeth and the unilateral scissor bite on the right side were successfully corrected (Figure 3). The pre-treatment and post-treatment lateral cephalographs and panoramic radiographs are presented in Figure 4 and Figure 5. The pre-treatment and post-treatment lateral cephalometric data are presented in Table 1.

## 4. Discussion

It has been reported that a scissor bite is a type of severe malocclusion with an estimated occurrence of 1.5%, and even fewer cases in primary dentition [4]. Early treatment is advocated because a spontaneous correction is unusual and the malpositioned condyles are allowed to sit in a bilaterally symmetric position with the treatment [3,17]. Previous studies or case reports have demonstrated that the enhancement of the early transverse development of a mandibular arch with fixed or removable appliances were effective ways to manage scissor bite [4,5].

In the present case, a prefabricated PFA (NOA™-F3) and a prefabricated oral screen (NOA™-T) were used. The NOA™-F3 is a Frankel-III-shaped functional appliance, which was used to induce the mandible to move backward and relieve the occlusion discrepancy of the dental arch. Compared with traditional functional appliances, PFAs might be more cost effective. With both functional appliance and positioner characteristics, PFA treatment showed some clinical advantages such as comfort in wearing, and correcting the skeletal and dental problems simultaneously [7,10]; however, there were some disadvantages of PFA treatment which should be noted. As most of PFAs are created with soft silicone and might be easy to be break during the treatment, treatment compliance needs to be carefully evaluated and only the patient with good compliance might be able to achieve favorable results. Moreover, there were evidences suggesting that other types of functional appliances might give better treatment results in a comparable time frame [6,7,8,9].

The treatment mechanisms of NOA-F3 for an anterior crossbite were to retrocline the lower anterior teeth, procline the upper incisors, guide the mandible backwards (Figure 1), and for the scissor bite to open the posterior deep bite with the occlusal plate, lingually moving maxillary posterior teeth with an elastic vestibular shield, buccally uprighting mandibular posterior teeth with the tongue, and guide the mandible to the left side and establish a proper posterior occlusion. In addition, a myofunctional exercise to break the tongue thrusting habits and reduce asymmetric muscular activities with NOA™-T, which was modified from an oral screen appliance. According to the basic of orofacial myofunctional therapy, it includes the treatment of facial muscle imbalances, training of the tongue posture, and establishing an equilibrium between the tongue, lip, and cheek muscles [18,19,20].

The oral rehabilitation of this case consisted of employing multiple resin-filled celluloid crowns on anterior teeth and the indirect-filled technique with the composite resin (multiple resin composite provisional restorations) on posterior teeth [21]. Oral rehabilitation with composite-inclined planes may be favorable to correct an anterior dental crossbite, and the indirect-filled technique could be used to cover the carious lesions and to build-up the crown in the process of a scissor bite correction. In the present case, the provisional restorations were designed to offer bilateral contacts of all posterior teeth in centric relation with the harmonious in excursive movements according to the concept of a mutually protected occlusion.

The favorable outcome in this case could be attributed to the fact that malocclusion and imbalance in muscular activity were both targeted in the treatment plan, and an appropriate patient selection was also important as the patient’s cooperation played a key role in the removable functional appliance treatment [22]. Previous studies indicated that younger patients might have better treatment compliance as the non-compliance rate with Twin-block treatment was 16% at the age of 9.7 and 33.6% at the age of 12.4 [23,24]. Therefore, the interceptive treatment of severe malocclusions with removable appliances in early age might have a better chance to be successful.

## 5. Conclusions

This case report presented a new PFA system and treatment protocol in primary dentition which resulted in a favorable outcome, and the clinical effectiveness of this appliance system warrants further investigation.

## Figures and Tables

**Table 1:** The pre-treatment and post-treatment lateral cephalometric data.

Angular/Linear Measurement	Mean (6 Years)	Initial	Final
SNA (°)	81–83	82	82
SNB (°)	79–81	82	79
ANB (°)	2–4	0	3
Wits Appraisal (mm)	0–1	−4.5	−4
FMA (°)	22–28	23.6	30
IMPA (°)	87–89	88	81
U1 to NA (mm)	21–23	15	24
L1 to NB (mm)	24–26	20	16
NSAr (saddle angle) (°)	118–126	128	128
Gonial angle (°)	129.8	146	135
NSGn (°)	59.8	67	67
Y-axis (°)	53–66	61	66
Nasion perpendicular to point A (mm)	0–1.0	−2.7	−3.6
Upper pharyngeal space (mm)	15–20	6.4	6
Lower pharyngeal space (mm)	11–14	12.7	11.8

SNA: Sella-Nasion-A point, SNB: Sella-Nasion-B point, ANB: A point-Nasion-B point, FMA: Frankfort-mandibular plane angle, IMPA: Incisor to mandibular plane angle, UI-NA: Upper incisor to NA line, LI-NB: Lower incisor to NBline.

## Data Availability

The datasets generated during and/or analysed during the current study are available from the corresponding author on reasonable request.
